# Cystitis Cystica Leading to Stricture of the Internal Urethral Orifice

**DOI:** 10.7759/cureus.65352

**Published:** 2024-07-25

**Authors:** Risako Yagi, Daisuke Watanabe, Takashi Ujiie, Takahiro Yoshida, Akio Mizushima

**Affiliations:** 1 Department of Urology, Koto Hospital, Tokyo, JPN; 2 Department of Palliative Medicine, Juntendo University Graduate School of Medicine, Tokyo, JPN; 3 Department of Molecular and Cellular Therapeutics, Juntendo University Graduate School of Medicine, Tokyo, JPN

**Keywords:** trans urethral resection of bladder tumor (turbt), posterior urethral stricture, lower urinary tract obstruction, internal urethral orifice, cystitis cystica

## Abstract

Cystitis cystica is a relatively common chronic reactive inflammatory disease caused by chronic irritation of the bladder mucosa. It is broadly considered one of the classifications of proliferative cystitis. The predilection site is the bladder trigone area, which may present with symptoms such as frequent urination, hematuria, and lower abdominal discomfort; however, it rarely causes bladder outlet obstruction. We present the case of a 59-year-old male patient suffering from incomplete urinary retention due to internal urethral orifice obstruction resulting from cystitis cystica. Following transurethral resection, the patient's dysuria rapidly improved, and the tumor did not recur.

## Introduction

Cystitis cystica and von Brunn’s nests represent a continuum of proliferative or reactive changes of the urothelium. These changes are thought to be triggered by a chronic inflammatory process such as recurrent urinary tract infections [1]. The most common site is the bladder trigone area, and symptoms such as frequent urination, bladder irritation, hematuria, and lower abdominal discomfort may be present [2]. Bladder outlet obstruction, which is mainly a symptom of benign prostatic hyperplasia, causes decreased urinary output and urinary retention and is strongly associated with dysuria in men. To our knowledge, cystitis cystica rarely causes bladder outlet obstruction [2-5], leading to incomplete urinary retention. In this report, we describe a case of cystitis cystica arising at the level of the internal urethral orifice, resulting in severe dysuria due to bladder outlet obstruction.

## Case presentation

**Figure 1:**
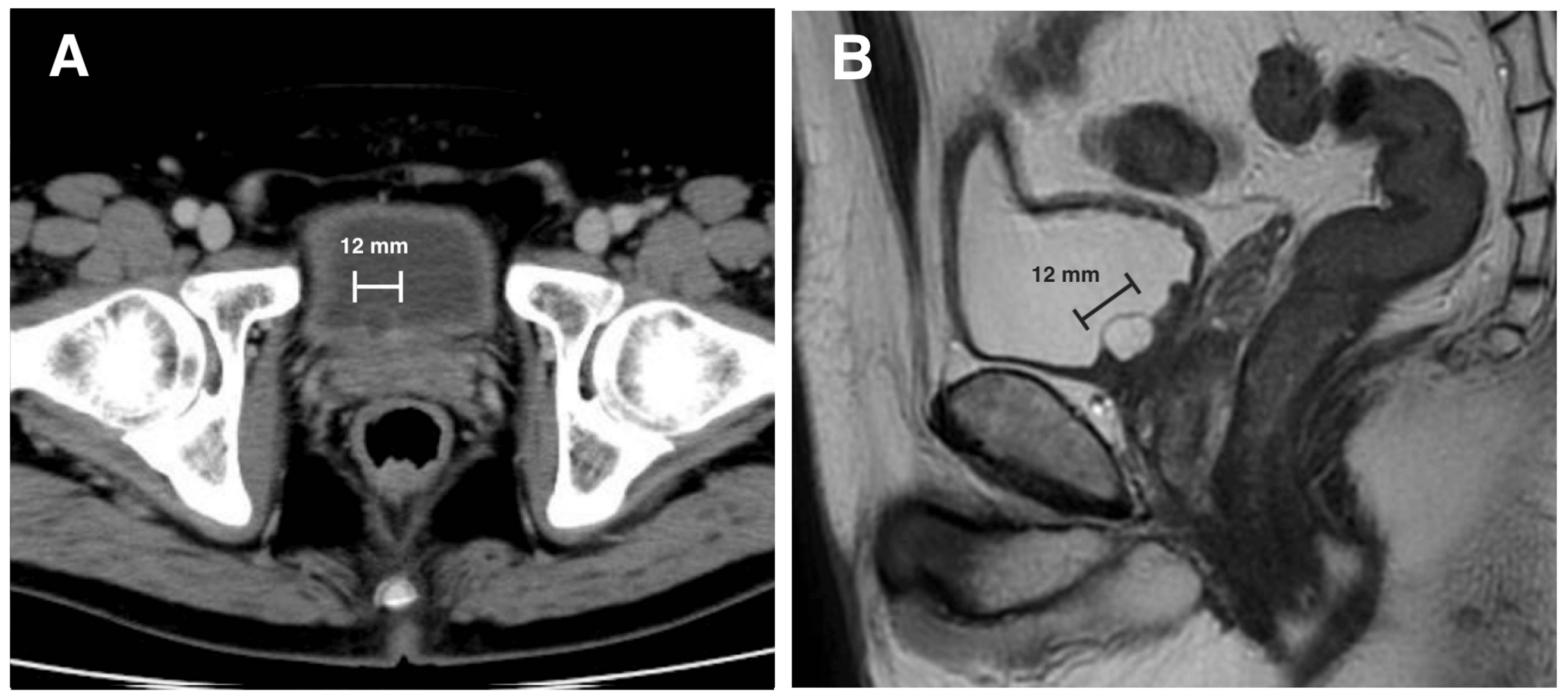
Axial contrast-enhanced computed tomography image (A) and sagittal magnetic resonance imaging T2-weighted image (B) of the bladder.

A 59-year-old man visited our department with complaints of strong residual urine sensation and urgency. A contrast-enhanced CT scan revealed a 12 mm-sized occupying lesion on the midline of the bladder neck (Figure 1), and the post-void residual urine volume was measured using a handheld bladder scanner and found to be 180 ml. The patient had no previous urologic history but had subjective symptoms of decreased urinary output for several years. Contrast-enhanced magnetic resonance imaging (MRI) revealed a cystic mass with a low signal on T1-weighted images and a high signal on T2-weighted images (Figure 1). No obvious enhancing component was identified. Cystoscopy was performed, and although there was no evidence of anterior urethral stricture or enlargement of the middle lobe of the prostate, a flat surface mass was found in the bladder neck.

**Figure 2:**
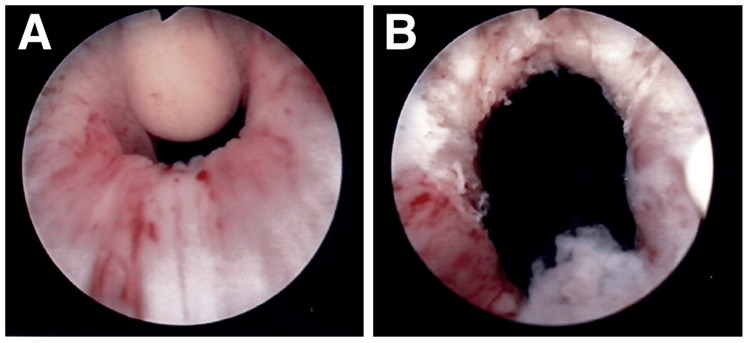
Intraoperative cystoscopy at the internal urethral orifice showing a cystic mass. A: endoscopic findings before resection; B: internal urethral orifice after tumor resection

**Figure 3:**
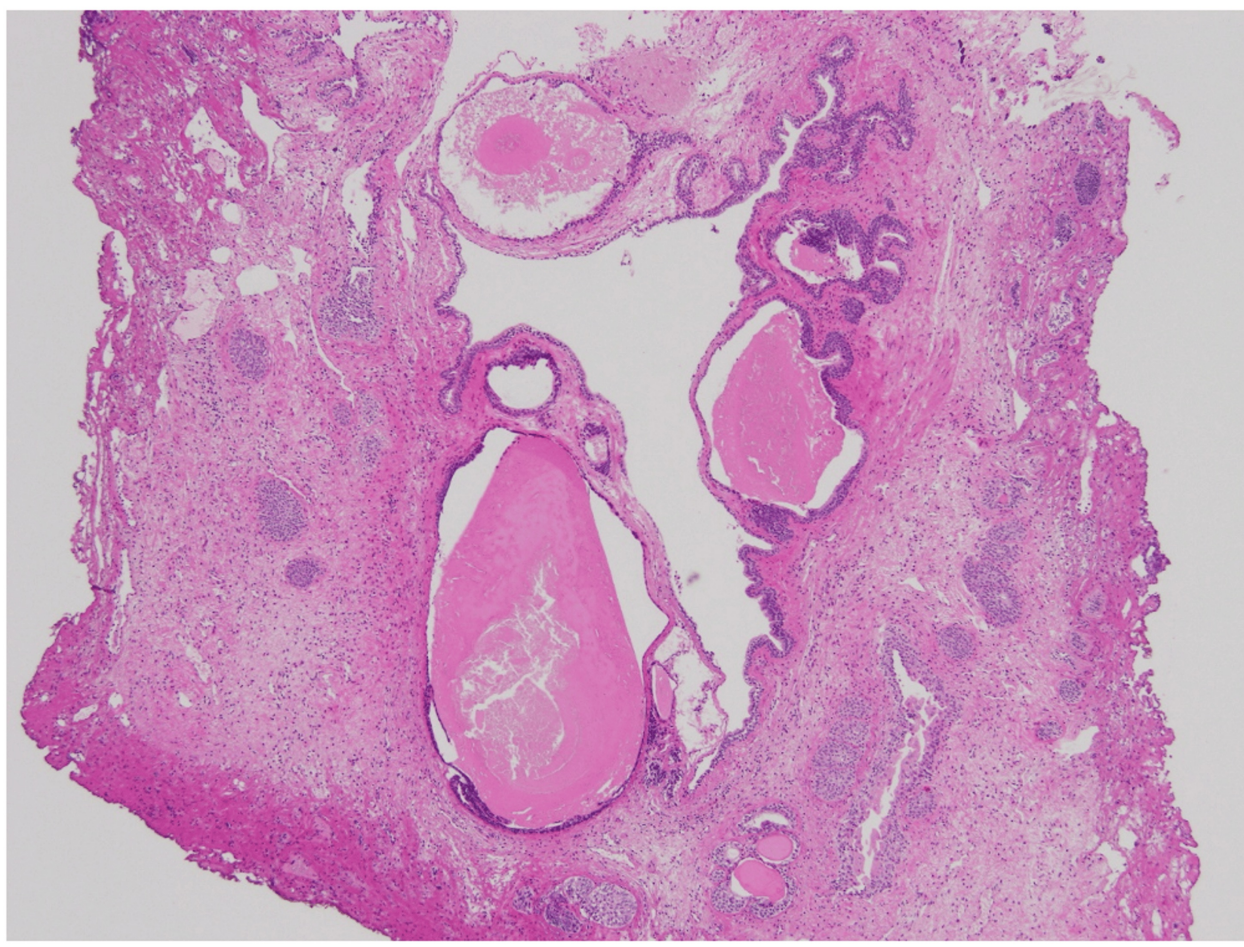
Pathological examination of the urothelium with cystitis cystica, with no evidence of carcinoma. Mangification x40, hematoxyline & eosine stain

When a transurethral resection was performed, the mass ruptured upon contact with a loop electrode, leaving only the mucosa (Figure 2). The site of the mass was excised, and the specimen was submitted to histopathology. Histopathological examination of the biopsy specimen showed urothelium exhibiting proliferative and cystic changes, including cystitis cystica. In addition, lymphocytic infiltration was detected in the surrounding interstitium (Figure 3). There were no malignant findings, and cystitis cystica was diagnosed. The patient's symptoms of decreased urinary output rapidly improved after the surgery, and no recurrence was observed six months after surgery.

## Discussion

Cystitis cystica is broadly defined as one of the classifications of proliferative cystitis. Proliferative cystitis is a localized collection of cells in the mucosal lining of the urothelium from the bladder [6]. Among proliferative cystitis, von Brunn’s nests, glandular cystitis, and cystitis cystica predominantly occur in the trigone to neck region of the bladder [1]. The frequency of cystitis cystica was 60 percent in the histopathological study of 100 grossly normal bladders obtained at postmortem examination [1]. Possible causes include mechanical irritation such as indwelling bladder catheters, stones, and urinary tract infection [1]. Patients with cystitis cystica often present with symptoms such as hematuria, pain during urination, frequent and residual urination, etc., because urinary tract infection is often present in the background [1,7]. In this case, there was no history of stones on examination by other departments, and the patient had no history of cystitis-like symptoms. No stones were found in the kidneys, ureters, or bladder on imaging, and the cyst obstructed the prostatic urethra to the bladder neck. The lesion caused incomplete urinary retention, and cystitis cystica was the cause of the patient's dysuria.

When cystoscopy is performed for diagnostic purposes, cystitis often reveals a single or multiple small submucosal translucent cysts. They may appear to be papillary tumors that are grossly suspicious of bladder cancer [8]. In the present case, the tumor was identified as a smooth mass with no surface irregularities, but it is impossible to determine whether it is benign or malignant with the naked eye. In clinical practice, transurethral resection of lesions in the urethra and bladder is necessary for diagnostic and therapeutic purposes. In this case, the internal urethral orifice was incompletely obstructed by a mass, and resection was necessary to ameliorate the patient's symptoms. Proliferative cystitis, including cystitis cystica, is not considered a precancerous lesion today [6]. Regardless of the pathological diagnosis, we believe that postoperative follow-up, especially cystoscopy, should be performed regularly in patients who have undergone transurethral resection. This is because we believe that it is useful not only to confirm whether the tumor has recurred but also to determine whether postoperative urethral stricture has occurred at the site of transurethral resection, which may cause urethral obstruction [9].

If the lesion is a mass large enough to obstruct the internal urethral orifice, as in the present case, it can be detected by abdominal imaging, but cystoscopy is the superior method for direct observation of the lesion on the mucosal surface and detection of abnormalities. All cases of urethral obstruction due to cystitis cystica that have been reported so far have been in relatively young men between the ages of one and 29 years old [2-5], and no reports have been found in elderly men. Mild to severe urethral obstruction due to benign prostatic hyperplasia is often seen in elderly men. Even though urethral obstruction caused by cystitis cystica in elderly men is uncommon, it may be worth bearing in mind that proliferative cystitis, including cystitis cystica, has a relatively high prevalence and may be one of the causes of urethral obstruction.

## Conclusions

A rare presentation of an elderly man with cystitis cystica that caused bladder outlet obstruction was reported. Unlike benign prostatic hyperplasia, cystitis cystica is not a common cause of urethral obstruction, but due to its high prevalence, it may be a disease that should be considered in the differential diagnosis of lower urinary tract obstruction in men.
